# Case report: Malignant epithelioid angiosarcoma in a Chinese female patient

**DOI:** 10.3389/fonc.2024.1398656

**Published:** 2024-08-16

**Authors:** Xiaohong Li, Lu Chen, Rong Ye, Chunyan Wu, Wenlei Zhuo

**Affiliations:** Department of Oncology, Second Affiliated Hospital of Army Military Medical University, Chongqing, China

**Keywords:** skin tumor, dermatology, dermatopathology, immunohistochemistry, PEComa

## Abstract

Perivascular epithelioid cell tumors (PEComas) are mesenchymal tumors that exhibit characteristic epithelioid or spindle cell morphology and typically grow around blood vessels. These tumors are characterized by the expression of melanocytic and smooth muscle markers, such as HMB-45, Melan-A, and smooth muscle actin, indicating a dual differentiation phenotype. PEComas are extremely rare diseases, and patients typically have a very poor prognosis. Here, we report a case of malignant cutaneous PEComa with pulmonary metastasis in a Chinese female and review relevant literature. The patient underwent surgical resection of a soft tissue tumor in the left upper arm under general anesthesia, and the subsequent pathological findings suggested a tumor with perivascular epithelioid cell differentiation (PEComa). The patient received adjuvant chemotherapy and radiotherapy after surgical resection, along with monitoring through computed tomography (CT) scans. Three months later, pulmonary metastasis was detected, but both the cutaneous PEComa in the left upper limb and the pulmonary metastatic lesions were stably controlled under active management and treatment. This is a rare case worth reporting and studying, and therefore, we conducted a long-term follow-up, and we hope to provide help for the clinical treatment of PEComa.

## Introduction

Perivascular epithelioid cell tumors (PEComas) are a rare type of mesenchymal tumor characterized by proliferation of perivascular epithelioid cells (1). They were first discovered in the early 1990s, these tumors exhibit a unique cell type that displays characteristics of both epithelial and smooth muscle cells, with typical epithelioid or spindle cell morphology and a tendency to grow around blood vessels (2–4). PEComas exhibit distinct phenotypes and immunohistochemical features, expressing markers of both melanocytic and smooth muscle differentiation (5). PEComas can occur in various organs throughout the body, including the uterus, kidneys, liver, lungs, gastrointestinal tract, pancreas, and soft tissues (6, 7). They can develop at any age but are more commonly observed in adults. PEComas can present as isolated tumors or as part of genetic syndromes such as tuberous sclerosis complex (TSC) or lymphangioleiomyomatosis (LAM) (8). PEComas have a very poor prognosis with no effective treatment, and conventional therapies are often ineffective, resulting in a survival period of only a few months. In this case, we firstly report a histopathologically confirmed malignant cutaneous PEComa with pulmonary metastasis in a Chinese female. After treatment, both the cutaneous PEComa in the left upper limb and the pulmonary metastatic lesions were stably controlled. Furthermore, in this case, the patient’s survival time is significantly longer, far exceeding previous reports.

## Case presentation

**Figure 1:**
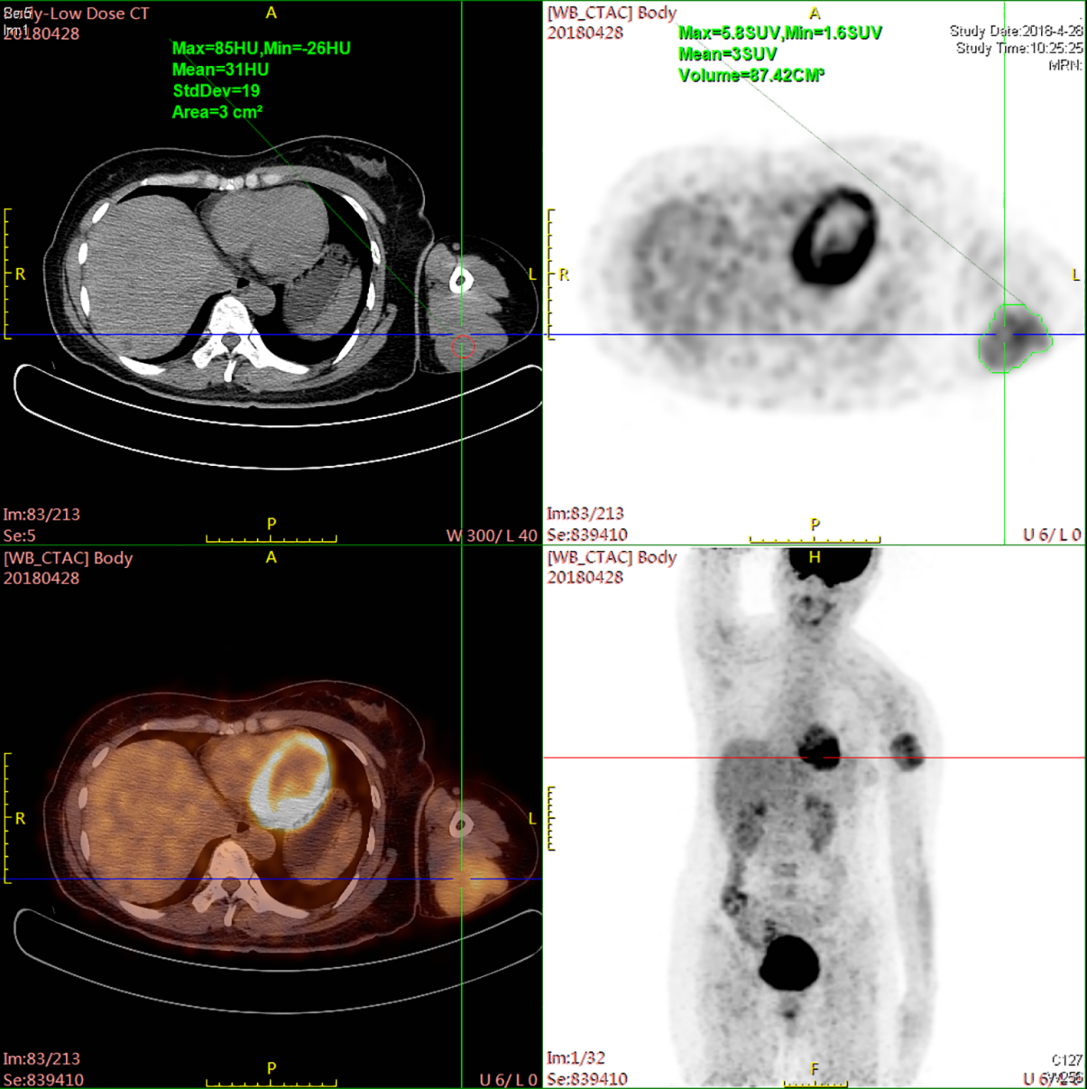
PET-CT scan revealing increased metabolic activity within a nodular soft tissue density located in the posterior subcutaneous area of the upper arm.

**Figure 2:**
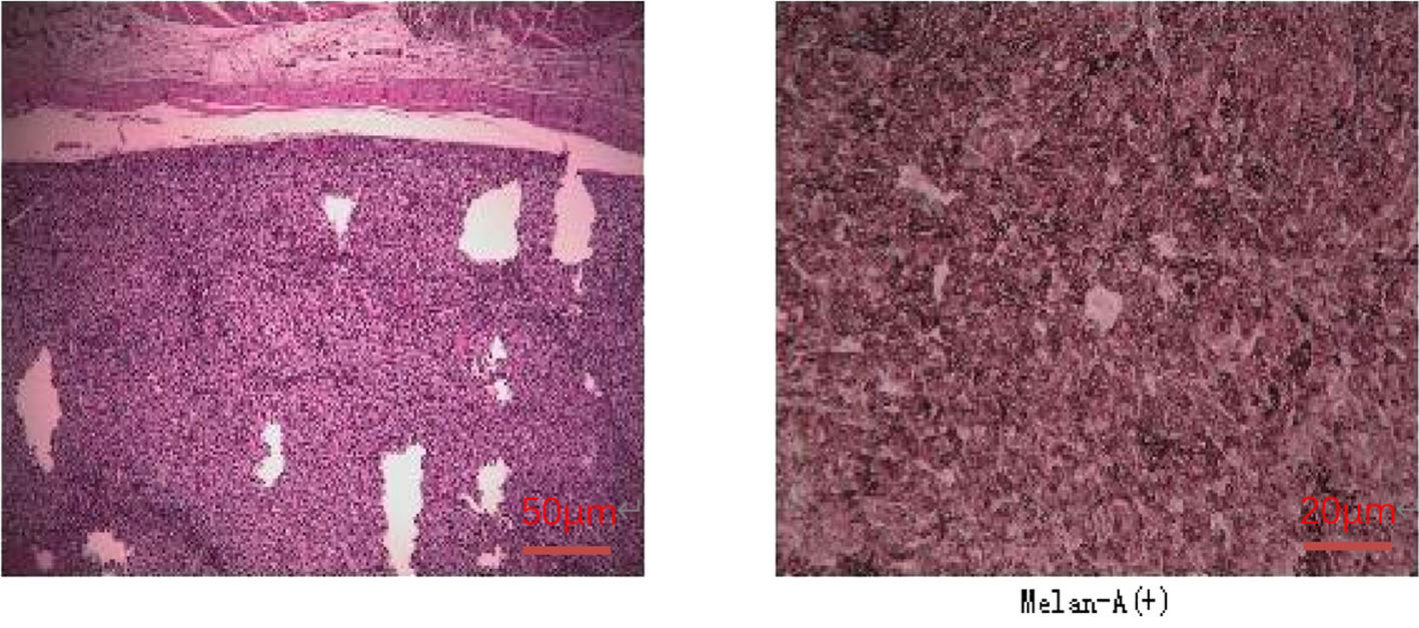
(A) HE and (A) Immunohistochemical staining.

**Figure 3:**
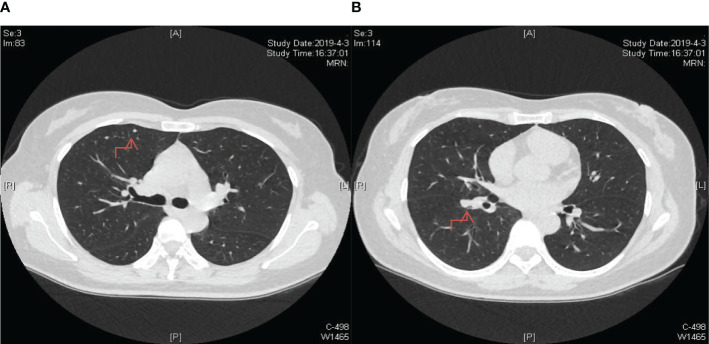
Post-completion of chemotherapy sessions, a follow-up chest CT scan revealed an augmented count and dimensions of nodules within bilateral lungs, indicating the emergence of lung metastases. **(A)** The lesion in the upper lobe of the right lung; **(B)** The lesion near the oblique fissure in the lower lobe of the right lung.

**Figure 4:**
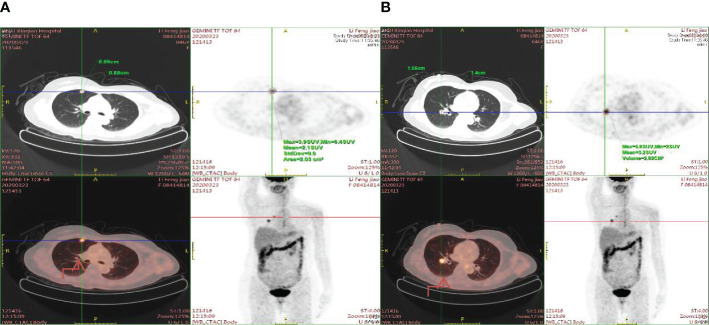
Enhanced radioactive uptake and a peak standardized uptake value (SUVmax) of 5.8 are observed in a larger nodule (1.06cm x 1.4cm) situated near the oblique fissure of the right lower lobe. **(A)** The nodule is situated in the right upper lung near the pleura. **(B)** The nodule is situated near the oblique fissure of the right lower lobe.

**Figure 5:**
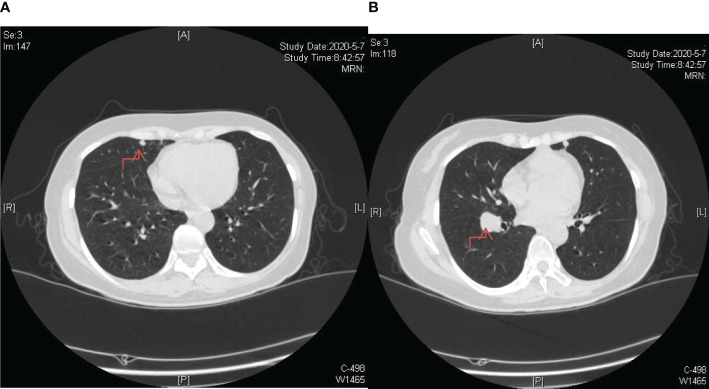
Enhanced nodule dimensions within the lung, with the largest measuring approximately 2.0 cm. **(A)** The lesion in the upper lobe of the right lung; **(B)** The lesion near the oblique fissure in the lower lobe of the right lung.

**Figure 6:**
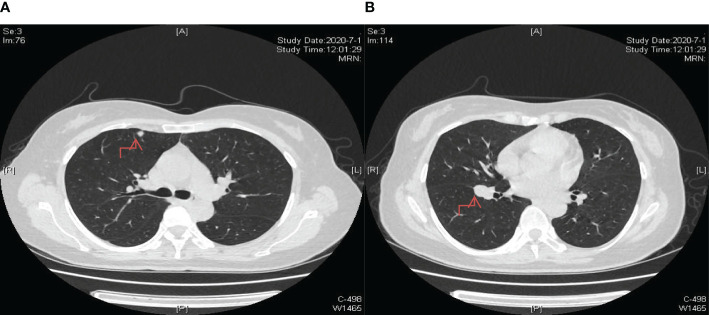
Significant reduction in lung nodule size observed after two cycles of chemotherapy incorporating a combination of 200mg sintilimab, 400mg albumin-bound paclitaxel, and 250mg apatinib as part of the treatment regimen. **(A)** The lesion in the upper lobe of the right lung; **(B)** The lesion near the oblique fissure in the lower lobe of the right lung.

**Figure 7:**
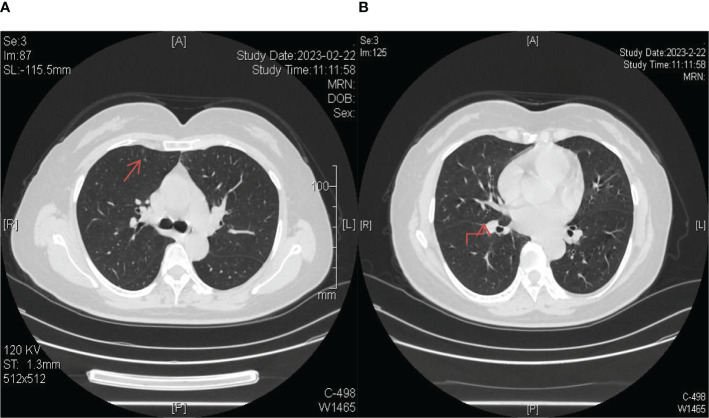
Longitudinal monitoring over a span of 3 years revealed consistent disease stability, as evidenced by regular chest CT scans. **(A)** The lesion in the upper lobe of the right lung; **(B)** The lesion near the oblique fissure in the lower lobe of the right lung.

A 47-year-old female patient without underlying diseases initially noticed a lump measuring 3cm*3cm on her left upper arm but did not pay much attention to it. Over the course of the following year, the lump increased in size and gradually became painful. As a result, she visited the hospital for consultation on March 27th, 2018. Physical examination revealed a firm, poorly mobile erythematous nodule measuring 3.5 centimeters on the left upper arm. Upon the doctor’s recommendation, the patient underwent ultrasound-guided biopsy of the lump on the left upper arm. Based on the morphological and immunohistochemical results, the initial diagnosis was soft tissue sarcoma. Additionally, detailed imaging studies were conducted. MRI findings showed a 6.4cm*5.5cm*4.0cm soft tissue mass in the posterior subcutaneous region of the upper arm. PET-CT indicated an elevated metabolic activity in the nodular soft tissue density in the posterior subcutaneous region of the upper arm (**Figure 1**). Following the doctor’s advice, the patient underwent surgical resection of the upper arm soft tissue tumor on April 28th, 2018. The pathology results confirmed the diagnosis of a perivascular epithelioid cell tumor (PEComa) in the upper arm. Immunohistochemical staining results showed: TFE-3 (focally weak+), MyoD1 (-), Myogenin (-), CK (-), CgA (-), SYN (locally +), S100 (-), CD34 (-), STAT6 (-), Desmin (-), EMA (+), Vimentin (+), Ki-67 (60–70%+), SF-1 (-), Actin (-), Melan-A (+), HMB45 (+), PAS staining (-) (**Figure 2**). At this point, chest CT indicated no evidence of lung metastasis, while brain MRI and whole-body bone scan showed no significant abnormalities. The patient underwent adjuvant chemotherapy with a regimen consisting of recombinant human endostatin (210mg) plus AP (pirarubicin 60mg and nedaplatin 120mg). Additionally, adjuvant radiotherapy was administered to the upper arm. Three months later, a follow-up MRI of the upper arm showed no signs of recurrence. However, a chest CT revealed the presence of small nodular lesions in the pleura of the left upper lobe anterior segment and the posterior segment of the left lower lobe. Subsequently, the patient underwent regular outpatient follow-ups to evaluate the effectiveness of the treatment plan. After completion of all chemotherapy sessions, a repeat chest CT scan on April 3th,2019. showed an increase in the number and size of nodules in both lungs, suggesting the presence of lung metastases (**Figures 3A, B**). The patient was prescribed anlotinib for oral treatment. During the following year, the patient underwent regular chest CT scans, which showed no significant enlargement of the lung metastatic lesions. It is suggested that anlotinib is effective in controlling the disease during this period on March 23th, 2020, a PET-CT scan revealed multiple nodular densities with increased uptake in both lungs, primarily distributed beneath the pleura. Some nodules showed adhesions to the pleura. A larger nodule measuring approximately 1.06cm*1.4cm was located adjacent to the oblique fissure of the right lower lobe, exhibiting increased radioactive uptake with a maximum standardized uptake value (SUVmax) of 5.8 (**Figures 4A, B**). It suggested that anlotinib alone was no longer effective at this time and could not control the progression of the disease. The patient’s chemotherapy regimen was adjusted to single-agent chemotherapy with gemcitabine (1.6g) on days 1 and 8, while continuing oral anlotinib treatment. Two months later, a repeat chest CT scan showed some of the nodules inside the lung have increased in size compared to before (the larger one is approximately 2.0cm), indicating Anlotinib combined with chemotherapy still cannot halt the progression of the disease. (**Figures 5A, B**). After assessing the patient’s condition, we have adjusted the treatment plan to include a combination of 200mg Sintilimab, 400mg albumin-bound paclitaxel, and 250mg apatinib for 2 cycles of chemotherapy. After two weeks of treatment with the new treatment plan, we observed a significant reduction in the size of the nodules within the lungs compared to before (**Figures 6A, B**). The treatment was deemed effective, and the patient continued with the 3rd and 4th cycles of chemotherapy. Maintenance treatment was continued with sintilimab plus apatinib. During the subsequent 3-year period, the patient underwent regular chest CT scans, and the disease remained stable (**Figures 7A, B**). Of note, the patient had another follow-up visit in December 2023, with the condition well controlled, and there were basically no lesions in the lungs.

## Discussion

Primary perivascular epithelioid cell tumor (PEComa) is a rare tumor that originates from perivascular epithelioid cells (9). Primary PEComas can occur at any age but are more commonly observed in adults. The average age of occurrence is between 30 and 50 years, although cases have been reported in children and older individuals as well (10). Furthermore, primary PEComas can arise in multiple organs, including the uterus, lungs, kidneys, gastrointestinal tract, and bladder (11). The symptoms and manifestations of PEComa depend on the location and size of the tumor. Some patients may have no symptoms, while others may experience organ-specific symptoms such as abdominal pain, abdominal mass, difficulty breathing, or urinary symptoms. Folpe et al. proposed a classification system for PEComas, in which a diagnosis of malignancy is determined by the presence of two or more high-risk features: size > 5 cm, infiltrative growth pattern, high nuclear grade and cellularity, mitotic rate > 1/50 high-power field (HPF), necrosis, and vascular invasion (12). These malignancy criteria were developed for PEComas originating from soft tissue and gynecologic sources; however, the data can be provisionally extrapolated to very rare cases involving the skin. The standard treatment for PEComa includes chemotherapy with Pazopanib, Temozolomide, and Imatinib. Guidelines also suggest the use of Sunitinib and Docetaxel, but their therapeutic efficacy is limited, leading to a shorter overall survival (13).The case we presented involves a rare occurrence of primary PEComa. In the initial stage of treatment, which includes surgery and postoperative radiation and chemotherapy, local disease control has been achieved. It is worth noting that during the course of treatment with postoperative chemotherapy and radiation therapy, the patient’s PEComa was controlled, but pulmonary metastasis occurred. For this pulmonary metastasis lesion, we found that changing the chemotherapy was ineffective, and the efficacy of Anlotinib alone was also limitative. Ultimately, with appropriate treatment, the pulmonary metastatic lesions were significantly controlled by combining Sintilimab with apatinib therapies.

The clinical presentation of perivascular epithelioid cell tumors (PEComas) varies from benign to malignant, and they may exhibit a tendency for local recurrence and metastasis in certain cases (14). The clinical manifestations of PEComas can vary depending on the location, size, and invasiveness of the tumor. Since PEComas can occur in various organs throughout the body, specific clinical features may differ based on the affected organ. Therefore, accurate diagnosis and appropriate risk stratification are necessary for optimizing management. Surgical resection is the mainstay of treatment, and the extent of surgery depends on the size, location, and malignant potential of the tumor (15, 16). Adjuvant therapies such as chemotherapy or targeted therapy may be considered in advanced or metastatic disease, although evidence of their effectiveness is limited (17). Due to the rarity of PEComas, there is limited data regarding optimal treatment and long-term prognosis. Collaborative efforts, such as international registries and clinical trials, are crucial for improving our understanding of these tumors and enhancing treatment outcomes for patients. Further research is needed to elucidate the underlying molecular mechanisms, identify potential therapeutic targets, and develop new treatment strategies for this intriguing group of tumors. As a result of the rarity of PEComas and limited data from clinical trials, the optimal chemotherapy regimen has not been firmly established. PEComas are generally considered resistant, indicating a poor response to traditional chemotherapy agents. However, in cases of advanced or metastatic PEComas where complete surgical resection is not feasible, chemotherapy may be considered as a palliative treatment option (18). The choice of chemotherapy regimen may vary based on individual patient factors, tumor characteristics, and institutional practices. Currently, there is no specific chemotherapy regimen that has been proven universally effective for PEComas. However, some commonly used chemotherapy drugs for the treatment of advanced or metastatic PEComas include mTOR inhibitors, as dysregulation of the mammalian target of rapamycin (mTOR) pathway is a characteristic feature of PEComas. Therefore, mTOR inhibitors such as sirolimus (rapamycin) and everolimus have been studied as potential targeted therapies (19–21). These drugs inhibit mTOR signaling and may provide some benefit in specific PEComas, particularly those associated with tuberous sclerosis complex (TSC) or showing activation of the mTOR pathway. Various chemotherapy regimens have been reported in the literature for advanced or metastatic PEComas, often employing combination drug therapies. These regimens may include gemcitabine, ifosfamide, dacarbazine, or anthracycline-based drugs (22, 23). However, the effectiveness of these regimens is mainly based on anecdotal reports and individual case studies, and their overall efficacy remains uncertain. It is worth noting that the response of PEComas to chemotherapy is unpredictable, and the benefits and risks of chemotherapy should be carefully evaluated on a case-by-case basis (24–26). Additionally, targeted therapies and immunotherapies are emerging as potential treatment options for PEComas, and clinical trials investigating novel agents are ongoing (27). Ultimately, the management of advanced or metastatic PEComas requires a multidisciplinary approach involving oncologists, surgeons, and other specialists. Treatment decisions should consider tumor characteristics, patient factors, potential side effects, and existing clinical evidence. Collaboration between clinical trials and medical centers is crucial for advancing our understanding of PEComas and determining effective treatment strategies.

The exact pathogenesis of primary perivascular epithelioid cell tumors (PEComas) is not fully understood (28). However, several proposed theories and molecular alterations are associated with the development of these tumors. Studies have indicated the involvement of the tuberous sclerosis complex (TSC) pathway in the pathogenesis of PEComas (29). A significant proportion of PEComas occur in patients with tuberous sclerosis complex (a hereditary disease caused by mutations in the TSC1 or TSC2 genes), which encode proteins involved in the regulation of the mammalian target of rapamycin (mTOR) pathway, playing a critical role in cell growth and proliferation (30). Dysregulation of the mTOR signaling due to functional mutations or loss of TSC1 or TSC2 leads to abnormal cell growth and the development of PEComas. Even in cases unrelated to tuberous sclerosis complex, activation of the mTOR pathway has been observed in some PEComas. Studies have shown increased expression of phosphorylated mTOR and its downstream targets, indicating dysregulated mTOR signaling. Abnormal activation of the mTOR pathway may be involved in the pathogenesis of PEComas through mechanisms independent of TSC gene mutations. mTOR inhibitors, including Sirolimus, Everolimus, and Temsirolimus, have been used in the treatment of PEComas, but the results are not very promising (31). The only FDA-approved treatment for advanced malignant PEComa in adults is nab-paclitaxel (albumin-bound nanoparticles of paclitaxel), based on the results of the AMPET clinical trial. The treatment has shown some progress, but it has its limitations. Unfortunately, for this case, the effectiveness of nab-paclitaxel is also not good, and it did not manage to control the progression of pulmonary metastases. Furthermore, genetic alterations have been identified in PEComas, although their significance and exact roles in tumor development are still under investigation. These alterations include chromosomal gains and losses, allelic losses in specific genomic regions, and rare somatic gene mutations such as TFE3 and CATSPERB (32, 33). However, it is important to note that these genetic alterations are not consistently observed in all PEComa cases, suggesting molecular heterogeneity within this tumor group (34). It is worth noting that some studies suggest potential influences of hormones or growth factors on the development of PEComas. For example, estrogen and progesterone receptors have been detected in certain uterine PEComas, suggesting a role of hormones in their pathogenesis (35, 36). Additionally, increased expression of vascular endothelial growth factor (VEGF) and platelet-derived growth factor (PDGF) receptors has been observed in some PEComas, indicating the potential involvement of these growth factors in promoting tumor growth and angiogenesis (4, 37). In conclusion, the pathogenesis of PEComas involves multiple factors, including signaling pathway dysregulation, genetic alterations, and potential influences of hormones or growth factors. Further research is needed to elucidate the complex molecular mechanisms underlying the occurrence and development of PEComas and to identify potential therapeutic targets for this rare tumor. Immunotherapy plays a crucial role in PEComa, and some have also applied PD-1 in the treatment of PEComa, but the results have not been very promising. However, according to the literature, the current efficacy of using PD-1 alone is relatively low (10–12). Currently, perivascular epithelioid cell tumors (PEComas) are believed to have the potential for metastasis, although their metastatic rate is generally lower compared to many other malignant tumors (38). Therefore, there are relatively few reports regarding metastatic primary PEComas, especially those successfully controlling metastatic lesions. The most common route of metastasis for PEComas is lymphatic spread, primarily to regional lymph nodes (39). Lymph node involvement in primary PEComas indicates a more aggressive behavior and poorer prognosis. PEComas can also spread through the bloodstream to distant organs and tissues (40). The most common sites of hematogenous metastasis include the lungs, liver, bones, and brain. Direct invasion can also lead to the dissemination of tumor cells beyond the primary site, increasing the risk of metastasis (41). However, this mode of metastasis is extremely rare. It is important to note that not all PEComas exhibit metastatic behavior, and the risk of metastasis may vary among different cases. Factors such as tumor size, histological characteristics, mitotic activity, and vascular or lymphatic invasion can influence the likelihood of metastasis and disease progression. Metastasis in PEComas is associated with a poorer prognosis as it indicates a more invasive tumor behavior. Early detection, accurate staging, and appropriate management, including surgical resection and adjuvant therapy, are important considerations in the management of metastatic PEComas (42, 43).In the presented case, the primary PEComas lesion has remained under control, suggesting that local treatments (including surgery and subsequent postoperative radiotherapy) are effective for local PEComas control. Although adjuvant chemotherapy was added postoperatively, lung metastasis still occurred several months later, suggesting the possibility of chemoresistance in this case. Therefore, we subsequently administered the small molecule TKI (anlotinib). Initially, the patient was prescribed oral anlotinib, and regular chest CT scans were performed in outpatient follow-ups over the course of a year, consistently showing no significant enlargement of the pulmonary metastatic lesions, indicating the multi-targeted anti-angiogenic small molecule TKI anlotinib is effective for treatment. However, after one year, further progression of the metastatic lesions was observed, may due to Anlotinib resistance.

Despite administering nab-paclitaxel promptly, the lung metastatic lesions have not only failed to diminish but have instead increased in both number and size. We suspect that the nab-paclitaxel is resistant. Considering, Apatinib and Anlotinib have different target points, even though Anlotinib failed, Apatinib might still be effective. Despite the genetic testing suggesting a disadvantage for the application of PD-1 antibodies, Apatinib has effects on improving the immune microenvironment. Therefore, we suspect that the combination of Apatinib with Anlotinib might be effective. We found that the combination of Apatinib with Anlotinib is indeed effective. Maintenance treatment with sintilimab and apatinib was continued for three years, and the patient’s condition remained stable during this period. Of note, the patient had another follow-up visit in December 2023, with the condition well controlled, and there were basically no lesions in the lungs. Given the limited reports on primary PEComas with lung metastasis, our case may provide insights for the development of treatment strategies for patients with primary PEComas and lung metastasis.

We conducted a detailed analysis of the reasons for the favorable treatment outcome in this case. Numerous studies have reported that angiogenesis plays a crucial role in the development and progression of PEComas (44). There is also research suggesting that anti-angiogenic drugs may be effective in treating PEComas (45). Therefore, we opted for an anti-angiogenesis therapy. Furthermore, to achieve a better treatment outcome, we integrated the results of genetic testing. In this case, the patient was PD-L1 positive, which led us to combine the use of PD-1, resulting in a favorable response. After the failure of the Anlotinib combined with PD-1 treatment regimen, we adjusted the treatment to combine Apatinib with PD-1. Anlotinib and Apatinib target different aspects of treatment, allowing for a timely shift to an alternative drug in the context of chemotherapy resistance (46). This approach continued to modulate the tumor microenvironment, greatly enhancing the effectiveness of PD-1 (47). Subsequent follow-up assessments showed that the patient’s CT scans over the past one to two years demonstrated a sustained reduction in tumor size, indicating the effectiveness of the treatment.

## Conclusion

PEComas represent an exceedingly rare disease characterized by significant treatment challenges and a bleak prognosis. In this case study, we illustrate the journey of a Chinese female patient diagnosed with PEComas who underwent surgical resection followed by adjuvant chemotherapy and radiotherapy. Despite these interventions, she encountered pulmonary metastasis. However, through a comprehensive treatment approach targeting both metastatic and non-metastatic lesions (utilizing Sunitinib in combination with Apatinib and albumin-bound paclitaxel), the patient’s condition stabilized, demonstrating notable improvement and effective disease control.

## Data Availability

The original contributions presented in the study are included in the article/supplementary material. Further inquiries can be directed to the corresponding author.
